# Exposure to maternal depressive symptoms in childhood and adolescent suicide-related thoughts and attempts: mediation by child psychiatric symptoms

**DOI:** 10.1017/S2045796018000847

**Published:** 2019-02-04

**Authors:** S. M. Goodday, S. Bondy, H. K. Brown, R. Sutradhar, A. Rhodes

**Affiliations:** 1 Department of Psychiatry, University of Oxford, Oxford, United Kingdom; 2 Department of Epidemiology, University of Toronto, Toronto, Canada; 3 Department of Psychiatry, University of Toronto, Toronto, Canada; 4 Department of Biostatistics, University of Toronto, Toronto, Canada; 5 Institute for Clinical Evaluative Sciences, Toronto, Canada; 6 McMaster University, The Offord Centre for Child Studies, Hamilton, Canada

**Keywords:** Adolescents, child psychiatry, mediation, prospective study, suicide

## Abstract

**Aims:**

The nature of the association between child psychiatric symptoms and adolescent suicide-related thoughts (SRT) and attempts (SA) remains unclear. Our objective was to assess whether child psychiatric symptoms from 6 to 10 years of age mediate the association between exposure to maternal depressive symptoms in childhood and offspring SRT and SA in adolescence.

**Methods:**

A population-based cohort study was constructed by linking all eight cycles from the National Longitudinal Survey of Children and Youth (NLSCY), a nationally representative Canadian panel survey conducted from 1994 to 2009. Self-reported maternal depressive symptoms were measured when offspring were between 0 and 5 years. Maternal-reported child psychiatric symptoms and psychiatric comorbid symptoms were measured from 6 to 10 years, and offspring self-reported SRT and SA were measured between 11 and 19 years. Indirect effects, the effect proportion mediated and their corresponding bootstrapped 95% confidence intervals (CI) were estimated.

**Results:**

Hyperactivity and inattention significantly mediated the association between maternal depressive symptoms in childhood and risk of both SRT and SA from 11 to 19 years, where approximately 60% (SRT 95% CI 23–94%; SA 95% CI 27–95%) of this association was explained by hyperactivity and inattention. Psychiatric comorbid symptoms also significantly mediated this relationship and accounted for 50% (95% CI 18–81%) of this association with SA.

**Conclusions:**

Targeting hyperactivity and inattention, and co-occurring psychiatric symptoms in offspring of depressed mothers could reduce risk of SRT, eventual SA and halt progression towards suicide. However, further understanding of comorbid psychiatric symptoms in childhood that most strongly predict adolescent SA is needed.

## Introduction

Suicide-related behaviour begins in adolescence, and is strongly associated with the progression to suicide (Bridge *et al*., 2006), exacting a heavy toll in young populations. Psychological autopsy studies report that approximately 90% of individuals who die by suicide had a prior psychiatric diagnosis (Cavanagh *et al*., 2003), and community-based surveys of younger populations (14–25 years) with suicide attempts (SA) report prior psychiatric disorders in approximately 90% of cases (Wunderlich *et al*., 1998). However, 40% of youth who die by suicide under age 16 years do not meet full diagnostic criteria for a psychiatric disorder (Bridge *et al*., 2006). The nature of the psychiatric disorder–suicide relationship remains ill-defined and only recently have suicide-related behaviours been recognised as separate from psychiatric illness (Oquendo and Baca-Garcia, 2014). Further, there is evidence that psychiatric symptoms, in part, explain associations between adverse early environments and SA risk (Fergusson *et al*., 2000; Wanner *et al*., 2012). Knowledge of other antecedent risk factors of both suicide-related behaviours and psychiatric symptoms and mediating pathways is needed to understand the aetiology of suicide-related behaviour onset to inform preventive strategies.

Exposure to maternal depression in childhood is a risk factor for a broad range of psychopathology in offspring occurring from early childhood to late adolescence (Goodman *et al*., 2011). Studies also support that maternal depression may be associated with offspring suicide-related behaviour in adolescence (Goodday *et al*., 2017), although the nature of this association is understudied. In our prior work, we have shown that girls exposed to maternal depressive symptom during the first decade of life are at an increased risk of incident and recurrent suicide-related thoughts (SRT) and SA between 11 and 25 years of age (Goodday *et al*., 2018). Hypothesised mechanisms linking maternal depression to offspring psychiatric problems in addition to or interacting with genetics surround disruptions in neuro-programming of important emotional systems during sensitive windows in childhood. These disruptions in turn can have lasting influences on emotional functioning evident as early as childhood, producing a psychological vulnerability (Vedhara *et al*., 2012). Specific psychiatric symptoms may reflect a vulnerable phenotype resulting from maternal depression that in turn predicts SRT and SA.

Evidence is conflicting pertaining to which type of psychiatric diagnosis and or symptoms is associated with SRT and SA. Trajectory-based studies have shown that externalising symptoms and depression at age 11–12 years predict high-risk trajectories of SRT up to age 15 years (Adrian *et al*., 2016), and anxiousness and disruptiveness in kindergarten children predict SA between 15 and 24 years (Brezo *et al*., 2008). Externalising disorders may be more strongly related to SA, primarily through impulsivity (Verona, 2000), while internalising disorders may be more related to SRT via the symptom of hopelessness (Verona *et al*., 2004). In support of this, there is evidence that attention-deficit hyperactivity disorder at age 15 years is associated with SA but not SRT at age 16 (Hammerton *et al*., 2015), and genetic studies also support that the transmission of SA risk but not SRT is mediated by genetic transmission of impulsive–aggressive traits (Brent and Melhem, 2008). A common finding across studies is that psychiatric comorbidity (co-occurring psychiatric diagnoses) appears to heighten risk of both SRT and SA in adolescence and young adulthood (Lewinsohn *et al*., 1995; Wunderlich *et al*., 1998; Vander Stoep *et al*., 2011), where higher numbers of psychiatric diagnoses together predict a greater risk, compared with individual diagnoses alone. There is also some evidence that these associations are sex/gender-specific, however research is limited, particularly in younger ages (Rhodes, 2014*b*).

Few studies have specifically tested whether child or adolescent psychiatric symptoms mediate the association between exposure to maternal depression and SRT and SA (Goodday *et al*., 2017). Hammerton *et al*. (2015) reported that symptoms of major depression, generalised anxiety disorder and disruptive behaviour disorder, but not attention-deficit hyperactivity disorder at age 15 significantly mediated the association between chronic and severe, and even moderate maternal depression from 0 to 11 years of age and offspring SRT at age 16. In the same study, the same set of psychiatric symptoms in addition to attention-deficit hyperactivity disorder were found to mediate the association between maternal depression and offspring SA at age 16 (Hammerton *et al*., 2015).

Knowledge of pathways to incident SRT and SA could be useful for understanding their aetiology and for informing the development and implementation of preventive interventions. Our objective was to test whether different child psychiatric symptoms from 6 to 10 years of age mediate the association between exposure to maternal depressive symptoms from 0 to 5 years of age and adolescent SRT and SA from 11 to 19 years of age, using prospectively captured data from the National Longitudinal Survey of Children and Youth (NLSCY).

## Methods

### Data source and study population

The NLSCY was a nationally representative longitudinal survey with a maximum of 8 cycles, spaced every 2 years, conducted by Statistics Canada. The sampling frame was based on the Labor Force Surveys, which includes approximately 97% of the Canadian population excluding full-time members of the Canadian Armed Forces, inmates of institutions and residents of Indian reserves, Yukon, Nunavut and the Northwest Territories (Statistics Canada, 2007). Children (herein referred to as offspring) and the parent respondent of each participating household were followed from 1994 to 2009 including respondents between 0 and 11 years of age at baseline (cycle 1) and up to age 25 years by the last cycle. In over 90% of cases, the parent respondent was the biological mother and hereafter is referred to as the mother (Statistics Canada, 2007). All NLSCY cycles (1 to 8) were linked using unique personal identifiers.

**Fig. 1.**
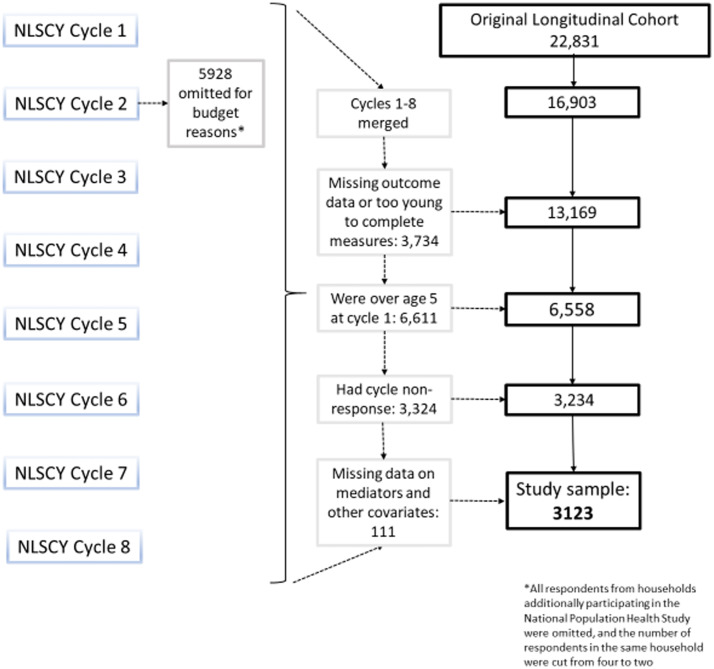
Flow chart of study sample selection.

This study included offspring who participated in the original NLSCY longitudinal cohort, which was comprised of 22 831 children, first sampled between 1994 and 1995. Due to Statistics Canada budget cuts and to reduce respondent burden on families, 26% of the baseline longitudinal sample were randomly excluded after cycle 1 (Fig. 1). However, the remaining sample accurately reflected the survey population (*n*  =  16 903) (Statistics Canada, 2007). For this analysis, the sample was reduced to offspring with outcome data, no cycle non-response and to those offspring who were between 0 and 5 years of age at baseline. The resulting study population included 3123 offspring between 0 and 19 years of age (Fig. 1). The proportion of missing data that reflected ‘not stated’ for the exposure, mediators and other covariates was low (<5%).

### Procedure

The NLSCY included both maternal- and offspring-reported measures conducted through computer-assisted interviews by Statistics Canada trained personnel or through school self-report questionnaires. The mothers of respondents were interviewed either in person by these personnel or by phone if this was not possible, while youth offspring between 11 and 17 years completed self-reported measures in schools. When offspring reached 18 years of age, the youth survey was completed by computer-assisted phone interviews to accommodate the ageing cohort and capture those no longer in school. Informed consent was completed by young adult respondents when completing computer-assisted interviewing, while the mother completed informed consent for their children and adolescents in the study (Statistics Canada, 2007). This study was approved by the Research Ethics Board at the University of Toronto (#00032787).

### Measures

Specific time periods of measurement were chosen to ensure temporal order between the exposure, mediator and outcomes. The exposure was self-reported by the mother, the mediators were reported by the mother for her offspring in the survey, and the outcome was self-reported by the offspring.

Exposure to maternal depressive symptoms when offspring were between 0 and 5 years of age was the main exposure. Depressive symptoms were measured from the 12-item short form of the Center for Epidemiologic Studies Depression Scale (CES-D) (Boyle *et al*., 1993*a*) designed to assesses the frequency and severity of past-week depressive symptoms (Cronbach's *α* = 0.86) (Statistics Canada, 2009). The NLSCY is a panel study with different numbers of cycle participation and ages at each cycle. Given that mothers had different numbers of cycle participation and to reduce risk of selection bias where offspring with mothers responding to more cycles would have a greater likelihood of being exposed, total scores were averaged across repeated assessments accounting for number of cycles participated in. Further, given that these data are from a general population sample, there were considerable floor effects in several measures of psychiatric symptoms making the examination of continuous scores challenging. Therefore, average scores were coded over an established cut-off of nine to reflect possible cases of maternal depression (Somers M and Willms J, 2002).

SRT and SA occurring between 11 and 19 years of age were our main outcomes. SRT and SA were measured by asking offspring: ‘During the past 12 months, did you seriously consider attempting suicide?’ and ‘During the past 12 months, how many times did you attempt suicide?’ (Statistics Canada, 2007). Both SRT and SA measures were coded as binary variables indicating yes or no.

Child psychiatric symptoms, when offspring were between 6 and 10 years of age, were examined as mediators. These measures were derived from the NLSCY parent-reported behaviours scale for 4–11 years old, designed to assess psychiatric symptoms that map onto Diagnostic and Statistical Manual-III – Revised and IV criteria (Statistics Canada, 2007). These maternal-reported scales were originally derived from the Child Behavior Checklist (Achenbach and Edelbrock, 1981). These measures have been shown to be internally consistent and valid (Boyle *et al*., 1993*b*; Boyle *et al*., 1993*a*). Examined subscales from the Behaviours Scale included emotion-anxiety (internalising), hyperactivity-inattention, conduct-physical aggression and indirect aggression. Total scores for each subscale were averaged across measurements when offspring were between 6 and 10 years of age and then coded to reflect an average score over the 75th percentile, suggestive of moderate symptoms (Colman *et al*., 2007). A measure reflecting psychiatric comorbid symptoms was also derived from these measures reflecting two or more subscales meeting the cut-off of >75^th^ percentile (where 1  =  two or more symptoms and 0  =  none).

Potential confounders were identified *a priori* from the literature relevant to the exposure–mediator, mediator–outcome and exposure–outcome level. These included maternal-reported offspring stressful life events occurring between 4 and 10 years of age (the age range when this measure was available), maternal and spouse binge drinking when offspring were between 0 and 10 years of age, socio-economic status (SES) measured at baseline (when offspring were between 0 and 10 years of age) and offspring sex (online Supplementary Table S1).

### Statistical analysis

Differences in descriptive characteristics between offspring exposed and non-exposed to maternal depressive symptoms were estimated using standardised differences. Standardised differences are invariant to sample sizes (Yang and Dalton, 2012) and can be interpreted as small (0.20), medium (0.50) and large (0.80) effect sizes (Cohen, 1988).

Methods outlined by Shrout and Bolger (2002) and others (Mackinnon *et al*., 2007; Mackinnon and Cox, 2012) guided the mediation approach (online Supplementary Methods). Unadjusted and adjusted odds ratios (OR) and 95% confidence intervals (CI) were estimated to quantify the magnitude of each path A (exposure–mediator), B (mediator–outcome), and C (total effect) (Step 1: online Supplementary Methods). Several mediation models were estimated, quantifying the mediating effect of each independent psychiatric symptom subscale and psychiatric comorbid symptoms on both outcomes separately. Specifically, unadjusted and adjusted *β*-coefficients (regression coefficients) and their corresponding 95% CIs, and *p*-values were estimated to quantify the indirect and direct effects through a series of univariate and multivariable logistic regression models (Step 2: online Supplementary Methods). Single mediation models were estimated because independent effects of psychiatric symptoms were expected (conditional on the exposure and outcomes), which could have different unmeasured confounders, and suppression effects (different signs in mediating paths) were not ruled out. All variables (exposure, mediator and outcomes) were binary in light of floor effects. All models were adjusted for the same potential confounders: offspring age in years at baseline, offspring stressful life event, SES, maternal and paternal binge drinking and offspring sex. Offspring sex was also included as an interaction term with the exposure to account for the heterogeneity in these associations between males and females (Rhodes, 2014*b*) as numbers were too low to stratify by sex. The effect proportion mediated (EPM; Shrout and Bolger, 2002) and corresponding 95% CIs were calculated where appropriate (indirect effects/direct effects  + indirect effects) to quantify the percent of the total effect that is accounted for by the pathway through the mediator (Step 3: online Supplementary Methods). To ensure all models included the same number of observations at the outset, individuals with missing data on the mediator variables (<5%) were excluded.

To reduce risk of bias in mediation models, we performed several sensitivity analyses testing for: (1) exposure–mediator interaction; (2) whether the exposure is related to mediator–outcome controlled confounding variables; and (3) whether adjusted *β*-coefficients differ in those with and without cycle non-response.

To account for the NLSCY's complex survey design, the longitudinal weights and bootstrap weights provided by Statistics Canada were used to calculate *β*-coefficients and variances using SAS survey procedures. The weights were used to produce unbiased estimates of the Canadian population and account for post-stratification and non-response (Statistics Canada, 2007). All analyses were performed using SAS software (version 9.4).

## Results

### Characteristics of the sample

This analysis included 3123 offspring with a weighted total of 816 140. Differences in sample characteristics in exposed and non-exposed offspring can be found in Table 1. Briefly, the proportion of females was similar in exposed (52%) and non-exposed (53%) offspring (standardised difference: 0.02). There was a higher proportion of exposed offspring coming from low SES households compared with non-exposed offspring (standardised difference: 0.31). Exposed offspring were more likely to have experienced a stressful life event between 4 and 10 years of age compared with non-exposed offspring (standardised difference: 0.19) (Table 1).

**Table 1.** Standardised differences between proportions of sample characteristics in offspring exposed and non-exposed to maternal depressive symptoms, weighted^a^ to reflect the Canadian general population

		Exposed %	Non-exposed %	S.D.^e^
Offspring sex	Male	48.27	47.22	
	Female	51.73	52.78	0.02
Socio-economic status^b^	1 – Lowest	14.25	4.91	0.31
	2	18.15	12.36	0.17
	3	29.81	25.98	0.09
	4	21.73	25.11	0.07
	5 – Highest	15.28	30.67	0.37
Maternal binge drinking >10 occasions (0–10 years)^c^	No	96.53	96.99	
	Yes	3.47	3.01	0.00
Spouse binge drinking >10 occasions (0–10 years)^c^	No	83.27	89.48	
	Yes	16.73	10.52	0.17
Offspring stressful life events (4–10 years)^d^	No	17.92	26.03	
	Yes	82.08	73.97	0.19
Hyperactivity	No	65.19	79.20	
	Yes	34.41	19.44	0.34
Internalising	No	68.22	81.83	
	Yes	31.31	16.86	0.34
Conduct	No	70.33	76.82	
	Yes	29.27	21.88	0.16
Indirect aggression	No	74.00	81.12	
	Yes	22.07	15.44	0.18
Psychiatric comorbidity	No	65.67	78.15	
	Yes	33.37	19.40	0.32

S.D., standardised difference.

a Inverse probability weights were used to produce estimates that accurately reflect the characteristics of the Canadian population in 1994/1995 (the baseline of the longitudinal cohort from the NLSCY), excluding full-time members of the Canadian Armed Forces, inmates of institutions and those residing (during the time of the survey) in Yukon, Nunavut, Northwest Territories and Indian reserves.

b Socio-economic status corresponding categories are presented in online Supplementary Table S2.

c Represents proportion of maternal and spouse binge drinking occasions over ten when offspring were between 0 and 10 years.

d Yes reflects any maternal report of child offspring experiencing a stressful life event from 4 to 10 years of age.

e Absolute values of 0.2  =  small, 0.5  =  medium and 0.8  =  large effect sizes (Cohen, 1988).

In total, 11.7% of the sample were exposed to maternal depressive symptoms between 0 and 5 years of age. Among offspring reporting SRT, 17.79% were exposed, while 13.38% were non-exposed. Among offspring reporting SA, 9.45% were exposed, while 6.07% were non-exposed.

### Quantifying exposure–mediator (A), mediator–outcome (B) and total effects (C)

**Fig. 2.**
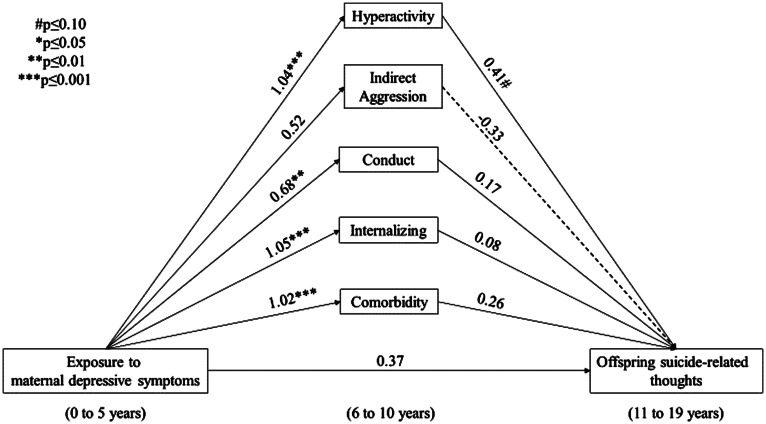
Adjusted (offspring age in years at baseline, offspring stressful life event (4–10 years), socio-economic status, maternal and paternal binge drinking (0–10 years), offspring sex, sex by exposure interaction) *β*-coefficients of the total effects, and exposure–mediator associations, and mediator–outcome associations with suicide-related thoughts as the outcome weighted (inverse probability weights were used to produce estimates that accurately reflect the characteristics of the Canadian population in 1994/1995 (the baseline of the longitudinal cohort from the NLSCY), excluding full-time members of the Canadian Armed Forces, inmates of institutions and those residing (during the time of the survey) in Yukon, Nunavut, Northwest Territories and Indian reserves) to reflect the Canadian general population.

**Fig. 3.**
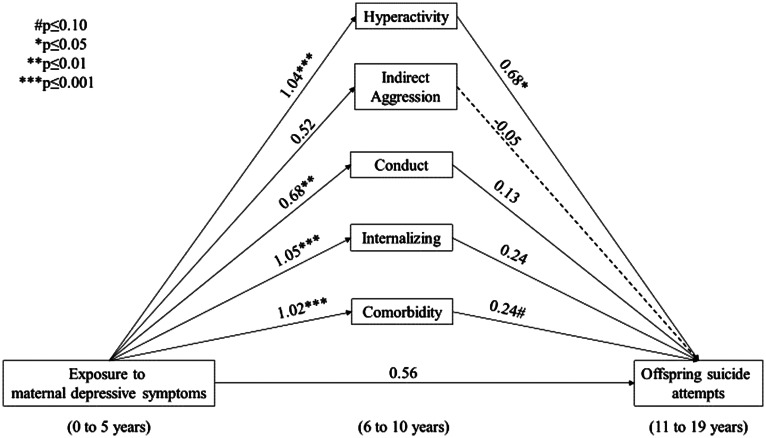
Adjusted (offspring age in years at baseline, offspring stressful life event (4–10 years), socio-economic status, maternal and paternal binge drinking (0–10 years), offspring sex, sex by exposure interaction) *β*-coefficients of the total effects, and exposure–mediator associations, and mediator–outcome associations with suicide attempts as the outcome, weighted (inverse probability weights were used to produce estimates that accurately reflect the characteristics of the Canadian population in 1994/1995 (the baseline of the longitudinal cohort from the NLSCY), excluding full-time members of the Canadian Armed Forces, inmates of institutions and those residing (during the time of the survey) in Yukon, Nunavut, Northwest Territories and Indian reserves) to reflect the Canadian general population.

Unadjusted paths A, B and C can be found in online Supplementary Figures S1 and S2. Exposure to maternal depressive symptoms occurring from 0 to 5 years of age statistically significantly increased the adjusted odds of internalising (OR 2.85, 95% CI 1.69–4.83), hyperactivity (OR 2.82, 95% CI 1.62–4.90), conduct (OR 1.97, 95% CI 1.17–3.31) and psychiatric comorbid symptoms (OR 2.77, 95% CI 1.61–4.74), but not indirect aggression (online Supplementary Table S2). None of the subscales statistically significantly increased the unadjusted or adjusted odds of SRT, while the adjusted odds of SA were statistically significantly elevated among offspring with hyperactivity compared with no hyperactivity (OR 1.97, 95% CI 1.09–3.54). The odds of SA were elevated in offspring with compared to without psychiatric comorbid symptoms, although this did not reach statistical significance (OR 1.61, 95% CI 0.94–2.76) (online Supplementary Table S3). The unadjusted and adjusted total effect (the association between exposure to maternal depressive symptoms and offspring SRT and SA) was not statistically significant, although in both cases was suggestive of an increased risk (SRT adjusted OR 1.45, 95% CI 0.84–2.51) and (SA adjusted OR 1.75, 95% CI 0.82–3.73), respectively (Figs 2, and 3).

### Mediation analyses

Table 2 presents the indirect effects, the EPM and their corresponding 95% CIs. The indirect effect through hyperactivity predicting SRT was statistically significant (*b*  =  0.43, 95% CI 0.30–0.55). Further, the indirect effect through hyperactivity predicting SA was statistically significant and larger in magnitude than SRT (*b*  =  0.70, 95% CI 0.54–0.87). The indirect effects through psychiatric comorbid symptoms predicting SA and SRT were also statistically significant. The indirect effects through internalising symptoms predicting SA but not SRT were statistically significant (*b*  =  0.25, 0.10–0.41), although lower in magnitude compared with paths through hyperactivity (Table 2). In total, 58% (95% CI 23–94%) and 61% (95% CI 27–95%) of the association between exposure to maternal depressive symptoms and offspring SRT and SA was accounted for by hyperactivity symptoms, while 50% (95% CI 18–81%) and 44% (95% CI 11–77%) of the association with SA and SRT was accounted for by psychiatric comorbid symptoms, respectively. Thirty-eight per cent of the association with SA was accounted for by internalising symptoms (95% CI 0.04–0.72) (Table 2).

**Table 2.** Adjusted^a^ direct and indirect effects and effect proportion mediated, weighted^b^ to reflect the Canadian general population

Outcome	Mediator	Total effect		Direct effect		Indirect effect		Proportion mediated (EPM)	
		*β*	95% CI^c^	*β*	95% CI^c^	*β*	95% CI^c^	EPM	95% CI^c^
Suicide-related thoughts	Hyperactivity/inattention	0.37	−0.17 to 0.92	0.31	−0.26 to 0.87	0.43	0.30–0.55	0.58	0.23–0.94
	Internalising	0.37	−0.17 to 0.92	0.37	−0.19 to 0.92	0.08	−0.04 to 0.20	0.18	−0.16 to 0.53
	Indirect aggression	0.37	−0.17 to 0.92	0.40	−0.16 to 0.96	−0.17	−0.30 to −0.03	—^d^	—^d^
	Conduct	0.37	−0.17 to 0.92	0.37	−0.17 to 0.92	0.11	0.00–0.22	0.23	−0.09 to 0.55
	Psychiatric comorbidity	0.37	−0.17 to 0.92	0.34	−0.21 to 0.89	0.26	0.15–0.37	0.44	0.11–0.77
Suicide attempts	Hyperactivity/inattention	0.56	−0.19 to 1.32	0.45	−0.35 to 1.25	0.70	0.54–0.87	0.61	0.27–0.95
	Internalising	0.56	−0.19 to 1.32	0.41	−0.2 to 1.32	0.25	0.10–0.41	0.38	0.04–0.72
	Indirect aggression	0.56	−0.19 to 1.32	0.56	−0.22 to 1.34	−0.03	−0.26 to 0.21	—^d^	—^d^
	Conduct	0.56	−0.19 to 1.32	0.56	−0.20 to 1.33	0.09	−0.06 to 0.24	0.14	−0.18 to 0.46
	Psychiatric comorbidity	0.56	−0.19 to 1.32	0.49	−0.30 to 1.27	0.48	0.33–0.63	0.50	0.18–0.81

a Offspring age in years at baseline, offspring stressful life event (4–10 years), socio-economic status, maternal and paternal binge drinking (0–10 years), offspring sex, sex by exposure interaction.

b Inverse probability weights were used to produce estimates that accurately reflect the characteristics of the Canadian population in 1994/1995 (the baseline of the longitudinal cohort from the NLSCY), excluding full-time members of the Canadian Armed Forces, inmates of institutions and those residing (during the time of the survey) in Yukon, Nunavut, Northwest Territories and Indian reserves.

c Estimated using Statistics Canada bootstrap weights.

d Effect proportion mediated was not calculated owing to evidence of suppression.

### Sensitivity analyses

Exposure–mediator interaction was not statistically significant (*p* > 0.05) for all subscales except for indirect aggression. Specifically, there was a statistically significant interaction between exposure to maternal depressive symptoms and indirect aggression (*p*  =  0.02), and this product term significantly contributed to the model predicting both SRT and SA according to the likelihood ratio test.

Unadjusted associations between exposure to maternal depressive symptoms and mediator–outcome controlled confounders were not statistically significant for maternal binge drinking (OR 1.21, 95% CI 0.55–2.66), although, just reached statistical significance for spouse binge drinking (OR 1.87, 95% CI 1.09–3.21), and offspring stressful life events (OR 1.61, 95% CI 1.02–2.55). However, unadjusted and adjusted path *β*-coefficients were similar (online Supplementary Figures S1 and S2).

In models including all offspring, irrespective of cycle non-response, *β*-coefficients became slightly stronger in magnitude and more statistically significant, as the sample size increased substantially (online Supplementary Figures S3 and S4). Indirect effects and EPMs for hyperactivity and psychiatric comorbid symptoms were similar (online Supplementary Table 4). Those with cycle non-response were less likely to come from households of high SES (standardised difference: 0.23) and were older age at cycle 1 (standardised difference: 0.21) and these covariates are adjusted for in all multivariable models (online Supplementary Table 5).

## Discussion

This study examined the mediating role of child psychiatric symptoms in the association between exposure to maternal depressive symptoms in childhood and adolescent SRT and SA using data from a Canadian population-based cohort. The largest EPMs were seen for symptoms of hyperactivity and inattention, accounting for approximately 60% of the associations between maternal depressive symptoms in childhood and offspring SRT and SA risk from 11 to 19 years of age. Psychiatric comorbid symptoms also mediated this relationship and accounted for 44 and 50% of this association with SRT and SA, respectively, while internalising symptoms accounted for 38% of this association with SA only. This study extends previous research by being prospective, establishing temporality, being nationally representative increasing external validity, and by clarifying the mediating role of child psychiatric symptoms occurring between 6 and 10 years of age specifically in comparison to other studies examining symptoms later in adolescence.

These findings are partially consistent with a study using data from the Avon Longitudinal Study finding that attention-deficit hyperactivity disorder at age 15 years mediated the association between maternal depressive symptoms occurring when offspring were 0–11 years of age and offspring self-reported SA at age 16 (Hammerton *et al*., 2015). This study also found that major depression, generalised anxiety disorder and disruptive behaviour disorder also mediated this association. We did not find evidence that conduct or indirect aggression were mediating pathways to SRT or SA. We examined psychiatric symptoms in childhood, not in adolescence and there may be differential effects by age (Costello *et al*., 2011). Collectively, these findings support that symptoms of attention-deficit hyperactivity disorder in childhood and adolescence partially explain the association between childhood exposure to maternal depression and offspring adolescent SA. These findings raise questions about the role of impulsivity in predicting SA. While there is debate as to the nature of the association between impulsivity and suicide-related behaviours (Klonsky and May, 2010), research has typically been conducted in older adolescents and adults.

Although exposure to maternal depression in childhood is associated with a broad range of psychopathology in childhood and adolescence (Goodman *et al*., 2011), further supported by this study, it appears as though there is specificity when it comes to which dimensions both indirectly or directly predict offspring SRT and SA. Studies linking specific psychiatric dimensions and diagnoses to suicide-related behaviour have yielded mixed findings and are heterogeneous by age. While we did not find that conduct or internalising subscales were associated with SRT or SA, in contrast to findings from other prospective studies of adolescents and young adults (Wunderlich *et al*., 1998; Vander Stoep *et al*., 2011; Adrian *et al*., 2016; Thompson and Swartout, 2018), this may reflect that in childhood these symptoms are less indicative of SA risk compared with adolescence, where these symptoms are more prevalent (Costello *et al*., 2011), and more proximal to the reported SA. Further, while depression is associated with SRT, some studies show that it is not associated with SA, and this is in keeping with theories surrounding the differential genetic transmission of suicide attempts in comparison to SRT (Brent and Melhem, 2008). Therefore, including measures of internalising symptoms of both depression and anxiety may wash out the effects of anxiety on SA risk, or depression on SRT risk. It remains unclear from our findings whether the mediating effect of internalising symptoms on SA risk was driven by depression or anxiety. More work is needed to unpack these groups of symptoms to understand which symptoms or clusters of symptoms are most predictive of SRT and SA.

What appears consistent across studies is that psychiatric comorbidity is associated with a greater risk of SA. Our findings support this observation, where psychiatric comorbid symptoms indirectly explained the association between maternal depressive symptoms and offspring SA. In fact, several studies have reported findings showing that psychiatric comorbidity is more strongly related to suicide-related behaviour compared with individual diagnoses alone (Lewinsohn *et al*., 1995; Wunderlich *et al*., 1998; Vander Stoep *et al*., 2011). This may reflect that greater severity of psychiatric illness, associated with higher psychiatric comorbidity, is more predictive of SRT and SA. Still, specific patterns of psychiatric comorbidity remain unclear.

### Strengths and limitations

This was a population-based study measuring symptoms, not diagnostic categories, and there are some potential threats to internal validity regarding information biases pertaining to the exposure and mediator variables. However, these findings are generalizable to the population, increasing external validity.

Maternal depressive symptoms and offspring psychiatric symptoms were reported by the mother. The CES-D is not a diagnostic tool, but rather a screening measure to identify possible cases of depression. On the other hand, this exposure is more reflective of maternal depressive symptoms seen in the general population. Maternal reports of child psychiatric symptoms have been shown to be rated more severe compared with youth reports (Kazdin *et al*., 1983; Treiber and Mabe, 1987). As such, there is a potential risk of differential misclassification of the mediator among exposed compared with non-exposed. However, there is inconsistent evidence that depressed mothers do perceive behaviour and emotional problems differently in their children compared with independent raters (Gartstein *et al*., 2009).

Outcomes were self-reported, which could be misreported relating to their sensitive nature. However, offspring were ensured confidentiality and anonymity when completing measures. Further, the method of interview (by phone *v*. in person) varied by the mothers of respondents. This information was not available; therefore, it is unknown how this may have impacted reporting. Mothers may have reported differently if interviewed in person owing to social desirability biases.

Even though this study was population-based, we were still restricted by low unweighted numbers owing to the rarity of outcomes and were unable to stratify by sex. These mediating effects are likely different in girls and boys. While we attempted to account for this heterogeneity by including sex and exposure interaction terms in our multivariable models, and it did improve their precision, we cannot speak to sex-specific intermediate pathways.

We selected the included confounders *a priori* and with the assumption that these variables are not caused by the exposure. The timing of variables was restricted based on what was available from the NLSCY. While there was not complete overlap between the exposure and life stress variable, given that depressive symptoms were stable over the first 5 years (Goodday *et al*., 2018), there are still instances where life stress in offspring could contribute to depressive symptoms in mothers. There are also likely other unmeasured confounders that we were unable to account for that differ by psychiatric symptoms. For example, physical and sexual abuses are strong predictors of adolescent SA (Rhodes, 2014*a*). We did not have a direct measure of abuse, although the life events measure used in the NLSCY included an item on abuse. We also did not have access to a measure of family history for suicide-related behaviour and were unable to account for genetic transmission of risk; still, studies have provided evidence of independent associations between maternal depression and offspring SA risk (Brent and Melhem, 2008; Hammerton *et al*., 2015).

Age when psychiatric symptoms were occurring could plausibly contribute to heterogeneity in the mediating effects examined. Given the panel design of the NLSCY, examining symptoms by each age would have not been feasible for everyone owing to the 2-year spacing of assessments. However, it was found that the rates of psychiatric symptoms from 6 to 10 years of age were quite stable (data not shown).

### Implications and conclusions

This research has implications for preventive and early intervention strategies for high-risk families where a mother is depressed. Further, assessing and appropriately treating hyperactivity and inattention symptoms, along with co-occurring psychiatric symptoms in offspring of depressed mothers could reduce risk of SRT, eventual SA and halt progression towards suicide. However, further understanding of the dimensional patterns of psychopathology in childhood that most strongly predict adolescent SA is needed. Family-based interventions and clinician monitoring targeting exposure to maternal depression when children are young aimed at increasing resiliency and reducing exposure during critical neurodevelopmental windows could have lasting influences on reducing vulnerability to psychiatric morbidity and SA risk (Zalsman, 2010). Finally, given adolescents of depressed mothers are an ultra-high-risk group for SRT and SA, close clinician monitoring of these individuals prior to and during the peak risk time for these thoughts and behaviours would be beneficial. Effectiveness of these suggested prevention strategies may vary by important differences in socio-demographic and healthcare system factors that warrant future research. The strongest risk factor for death by suicide, and future SA is a prior SA (Bridge *et al*., 2006); therefore, implementing preventive and early intervention strategies before youth reach crisis is crucial.

Symptoms of hyperactivity and inattention between 6 and 10 years of age explain 60% of the association between exposure to maternal depressive symptoms early in childhood and adolescent SA. Still, 40% of this association is accounted for by other factors that require further study. Categorical diagnoses likely do not tap into the complexity of psychopathologies that predict adolescent SA both indirectly via maternal depression and directly. More consideration of comorbid patterns of psychiatric symptoms is needed to fully understand this complex association.

## Supplementary material

For supplementary material accompanying this paper visit http://dx.doi.org/10.1017/S2045796018000847. click here to view supplementary material

## Availability of data

This study was a secondary analysis of data collected by Statistics Canada. As per Statistics Canada privacy and confidentiality guidelines, data cannot be released or shared with outside parties that have not applied for access through Canadian Research Data Centers.
