# Supplementary material for: Exposure to maternal depressive symptoms in childhood and adolescent suicide-related thoughts and attempts: mediation by child psychiatric symptoms
Source: Epidemiol Psychiatr Sci. 2019 Feb 4;29:e17. doi: 10.1017/S2045796018000847 (PMC8061164; doi:10.1017/S2045796018000847)
Supplement: Supplementary file 1 [file S2045796018000847sup001.docx]

Supplemental Methods. Mediation approach

**Mediation analytic procedure**

For each outcome (SRT or SA), multivariable logistic regression models were performed testing associations between exposure and mediator (A); mediator and outcome, controlling for exposure (B); exposure and outcome (C); exposure and outcome, controlling for mediator (C’), controlling for the same confounders in each model (Figure 1). Indirect effects were calculated by multiplying paths A and B.

*Figure 1. Mediation model*


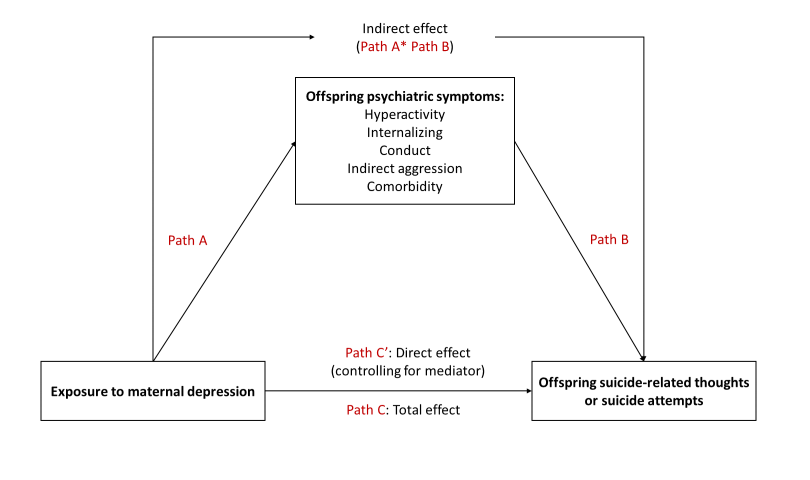


**Testing for presence of mediation**

*Step 1: Testing criteria for possible mediation*

Paths A, B and C are considered significant given the following accepted exemptions^1,2^:

A, B, or C is not significant but

- 1. the effect estimate is suggestive of an association
  2. there is sufficient evidence from the literature that an association exists
  3. the association between the exposure and outcome is distal

*Step 2: Testing if mediation is present*

Indirect effects are significant according to their bootstrapped 95% confidence intervals not spanning 0

*Step 3: Quantifying magnitude of mediating effects*

Calculate effect proportion mediated (EPM) and it’s corresponding bootstrapped 95% confidence intervals

EPM: Indirect effects / (Direct effect + Indirect effect) * 100

References:

1. MacKinnon DP, Fairchild AJ, & Fritz MS. Mediation analysis. Annual review of psychology. 2007;58, 593
2. Shrout PE, & Bolger N. Mediation in experimental and nonexperimental studies: new procedures and recommendations. Psychological methods. 2002 Dec;7(4):422.

Supplemental Table 1. Supplementary information on confounding variables

| Variable | Description | Coding | Rationale |
| --- | --- | --- | --- |
| Maternal/spouse binge drinking occasions | Average exposure to maternal/spouse binge drinking when offspring were between the ages of 0 to 10 years (cycles 1-6) | Average total number of past year binge drinking occasions (5 or more drinks on 1 occasion): Total occasions/number of cycles participated in  1=Average total occasions ≥10  0=Average total occasions <10 | The maternal and spouse binge drinking item was highly right skewed. This variable was dichotomized to above and below the 90^th^ percentile (10 binge drinking occasions) |
| Household SES continuous (used in multivariable models) | Composite score derived from maternal and spouse highest level of education, occupation and household income when offspring were between 0 to 10 years (Cycle 1) | Continuous score ranging from  -2.0 to 1.75  For descriptive purposes this score was categorized into the following according to Statistics Canada suggested categories^1^:  5=≥1.5  4=<1.5≥0.5  3=<0.5=>0.0  2=<0.0=>-0.5  1=<-0.5 | The SES composite score was not available in all NLSCY cycles and SES of families in childhood was the measure of interest |
| Offspring stressful life event | Offspring stressful life events (maternal reported in 4-10-year-olds, cycles 1-6): “Has ... ever experienced any event or situation that has caused him/her a great amount of worry or unhappiness?”. | 1 = Yes  0 = No | This variable was only measured in offspring =>4 years and was only derived from cycles 1 to 6 to capture the exposure time of interest (between 0 to 10 years of age) |
| Offspring Sex | Maternal reported sex of their child offspring (cycle 1) | 1=Male  0=Female | There was no measure of self-reported gender in the NLSCY |

SES: socio-economic status; NLSCY: National Longitudinal Survey of Children and Youth

References:

1. Statistics and Canada. National Longitudinal Survey of Children and Youth (NLSCY), Cycle 2 - Microdata User Guide: (2007, accessed May 15th, 2015).

Supplemental Table 2. Path A: Risk of child psychiatric symptoms from 6-10 years of age in offspring exposed versus non-exposed to maternal depression between 0-5 years of age, weighted^a^ to reflect the Canadian general population

|  | Unadjusted | | | Adjusted^b^ | | |
| --- | --- | --- | --- | --- | --- | --- |
|  | OR | 95%CI^c^ | | OR | 95%CI^c^ | |
| Internalizing | 2.23 | 1.53 | 3.25 | 2.85 | 1.69 | 4.83 |
| Hyperactivity | 2.15 | 1.50 | 3.09 | 2.82 | 1.62 | 4.90 |
| Indirect aggression | 1.57 | 1.02 | 2.41 | 1.68 | 0.97 | 2.90 |
| Conduct | 1.46 | 1.03 | 2.08 | 1.97 | 1.17 | 3.31 |
| Comorbidity | 2.05 | 1.38 | 3.03 | 2.77 | 1.61 | 4.74 |

CI: confidence interval; OR: odds ratio

^a^Inverse probability weights were used to produce estimates that accurately reflect the characteristics of the Canadian population in 1994/1995 (the baseline of the longitudinal cohort from the NLSCY), excluding full-time members of the Canadian Armed Forces, inmates of institutions, and those residing (during the time of the survey) in Yukon, Nunavut, Northwest Territories and Indian reserves

^b^offspring age in years at baseline, offspring stressful life event (4-10 years), socio-economic status, maternal and paternal binge drinking (0-10 years), offspring sex, sex by exposure interaction

^c^Estimated using Statistics Canada Bootstrap weights

Supplemental Table 3. Path B: Risk of suicide-related thoughts and attempt from 11-19 years of age in individuals with and without psychiatric symptoms from 6-10 years, weighted^a^ to reflect the Canadian general population

|  | Suicide-related thoughts | | | | | | Suicide-attempts | | | | | |
| --- | --- | --- | --- | --- | --- | --- | --- | --- | --- | --- | --- | --- |
|  | Unadjusted | | | Adjusted^b^ | | | Unadjusted | | | Adjusted^b^ | | |
|  | OR | 95%CI^c^ | | OR | 95%CI^c^ | | OR | 95%CI^c^ | | OR | 95%CI^c^ | |
| Internalizing | 1.17 | 0.78 | 1.77 | 1.08 | 0.70 | 1.67 | 1.39 | 0.79 | 2.43 | 1.27 | 0.68 | 2.12 |
| Hyperactivity | 1.49 | 0.99 | 2.24 | 1.51 | 0.98 | 2.32 | 1.88 | 1.09 | 3.26 | 1.97 | 1.09 | 3.54 |
| Indirect aggression | 0.81 | 0.52 | 1.27 | 0.72 | 0.44 | 1.16 | 1.11 | 0.50 | 2.45 | 0.95 | 0.41 | 2.21 |
| Conduct | 1.15 | 0.78 | 1.69 | 1.18 | 0.79 | 1.77 | 1.13 | 0.65 | 1.96 | 1.14 | 0.65 | 2.01 |
| Comorbidity | 1.36 | 0.91 | 2.03 | 1.30 | 0.86 | 1.95 | 1.74 | 1.01 | 3.02 | 1.61 | 0.94 | 2.76 |

CI: confidence interval; OR: odds ratio

^a^Inverse probability weights were used to produce estimates that accurately reflect the characteristics of the Canadian population in 1994/1995 (the baseline of the longitudinal cohort from the NLSCY), excluding full-time members of the Canadian Armed Forces, inmates of institutions, and those residing (during the time of the survey) in Yukon, Nunavut, Northwest Territories and Indian reserves

^b^Offspring age in years at baseline, offspring stressful life event (4-10 years), socio-economic status, maternal and paternal binge drinking (0-10 years), offspring sex, sex by exposure interaction

^c^Estimated using Statistics Canada Bootstrap weights

Supplemental Table 4. **Sensitivity analysis:** Adjusted^a^ direct and indirect effects and effect proportion mediated including offspring with cycle non-response, weighted^b^ to reflect the Canadian general population

|  |  | Indirect effects | 95%CI^c^ | | Effect proportion mediated | 95%CI^c^ | |
| --- | --- | --- | --- | --- | --- | --- | --- |
| Suicide-related thoughts | Hyperactivity | 0.43 | 0.37 | 0.49 | 0.55 | 0.26 | 0.84 |
|  |  |  |  |  |  |  |  |
| Suicide attempts | Hyperactivity | 0.71 | 0.63 | 0.79 | 0.65 | 0.37 | 0.94 |
|  | Comorbidity | 0.58 | 0.50 | 0.65 | 0.62 | 0.35 | 0.88 |

CI: confidence interval

^a^Offspring age in years at baseline, offspring stressful life event (4-10 years), socio-economic status, maternal and paternal binge drinking (0-10 years), offspring sex, sex by exposure interaction

^b^Inverse probability weights were used to produce estimates that accurately reflect the characteristics of the Canadian population in 1994/1995 (the baseline of the longitudinal cohort from the NLSCY), excluding full-time members of the Canadian Armed Forces, inmates of institutions, and those residing (during the time of the survey) in Yukon, Nunavut, Northwest Territories and Indian reserves

^c^Estimated using Statistics Canada Bootstrap weights

Supplemental Table 5. **Sensitivity analysis:** Standardized differences between covariates in offspring with and without cycle non-response, weighted^a^ to reflect the Canadian general population

|  |  | CNR Yes^f^ | CNR No^g^ |  |
| --- | --- | --- | --- | --- |
|  |  | %^h^ | %^h^ | SD^e^ |
| Sex | Male | 46.95 | 52.65 |  |
|  | Female | 53.05 | 47.35 | 0.11 |
|  |  |  |  |  |
| Socio-economic status^b^ | 1 - lowest | 10.83 | 6.01 | 0.17 |
|  | 2 | 17.92 | 13.04 | 0.14 |
|  | 3 | 28.88 | 26.43 | 0.05 |
|  | 4 | 22.54 | 24.71 | 0.05 |
|  | 5 - highest | 19.20 | 28.87 | 0.23 |
|  |  |  |  |  |
| Suicide attempt | no | 94.53 | 93.53 |  |
|  | yes | 5.47 | 6.47 | 0.04 |
| Suicide-related thoughts | no | 90.50 | 86.11 |  |
|  | yes | 9.50 | 13.89 | 0.14 |
|  |  |  |  |  |
| Maternal binge drinking >10 occasions (0-10 years)^c^ | no | 96.28 | 96.93 |  |
|  | yes | 3.72 | 3.07 | 0.04 |
| Spouse binge drinking >10 occasions (0-10 years)^c^ | no | 91.44 | 88.75 |  |
|  | yes | 8.53 | 11.25 | 0.09 |
|  |  |  |  |  |
| Offspring stressful life event (4-10 years)^d^ | no | 28.68 | 25.09 |  |
|  | yes | 71.27 | 74.91 | 0.08 |
|  |  |  |  |  |
| Exposure to maternal depression (0-5 years) | no | 82.11 | 88.31 |  |
|  | yes | 17.89 | 11.69 | 0.18 |
|  |  |  |  |  |
| Age at cycle one in years | 0 | 11.30 | 17.12 | 0.17 |
|  | 1 | 11.55 | 17.51 | 0.17 |
|  | 2 | 16.43 | 17.16 | 0.02 |
|  | 3 | 15.13 | 17.78 | 0.07 |
|  | 4 | 22.64 | 15.51 | 0.18 |
|  | 5 | 22.95 | 14.92 | 0.21 |
|  |  |  |  |  |
| Internalizing | no | 75.40 | 80.24 |  |
|  | yes | 18.61 | 18.55 | 0.00 |
|  |  |  |  |  |
| Hyperactivity | no | 76.32 | 80.29 |  |
|  | yes | 15.77 | 16.22 | 0.01 |
|  |  |  |  |  |
| Indirect aggression | no | 69.27 | 77.56 |  |
|  | yes | 24.64 | 21.19 | 0.08 |
|  |  |  |  |  |
| Conduct | no | 74.49 | 76.06 |  |
|  | yes | 19.56 | 22.74 | 0.08 |
|  |  |  |  |  |
| Psychiatric comorbidity | no | 70.67 | 76.69 |  |
|  | yes | 22.50 | 21.03 | 0.06 |

CNR: cycle non-response; SD: standardized difference

^a^Inverse probability weights were used to produce estimates that accurately reflect the characteristics of the Canadian population in 1994/1995 (the baseline of the longitudinal cohort from the NLSCY), excluding full-time members of the Canadian Armed Forces, inmates of institutions, and those residing (during the time of the survey) in Yukon, Nunavut, Northwest Territories and Indian reserves

^b^Socio-economic status corresponding categories are presented in supplementary table 1

^c^Represents proportion of maternal and spouse binge drinking occasions over ten when offspring were between 0-10 years.

^d^Yes: Any maternal report of child distressing stress from 4-10 years of age

^e^Absolute values of 0∙2=small, 0∙5=medium, and 0∙8=large effect sizes

^f^Offspring with one or more instance of cycle non-response

^g^Offspring with complete follow-up

^h^Some frequencies do not amount to 100 owing to missing data on specific covariate

Supplemental Figure 1. Unadjusted beta coefficients of the total effects, and exposure, mediator associations, and mediator, outcome associations with suicide-related thoughts as the outcome, weighted^a^ to reflect the Canadian general population


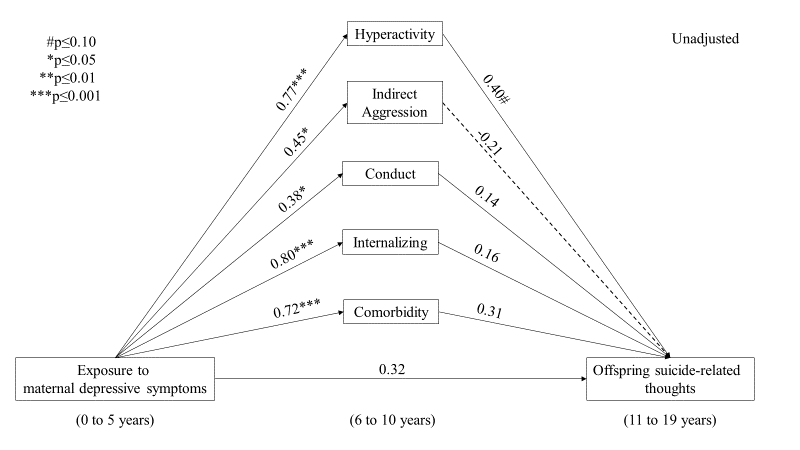


^a^Inverse probability weights were used to produce estimates that accurately reflect the characteristics of the Canadian population in 1994/1995 (the baseline of the longitudinal cohort from the NLSCY), excluding full-time members of the Canadian Armed Forces, inmates of institutions, and those residing (during the time of the survey) in Yukon, Nunavut, Northwest Territories and Indian reserves

Supplemental Figure 2. Unadjusted beta coefficients of the total effects, and exposure, mediator associations, and mediator outcome associations with suicide attempts as the outcome, weighted^a^ to reflect the Canadian general population


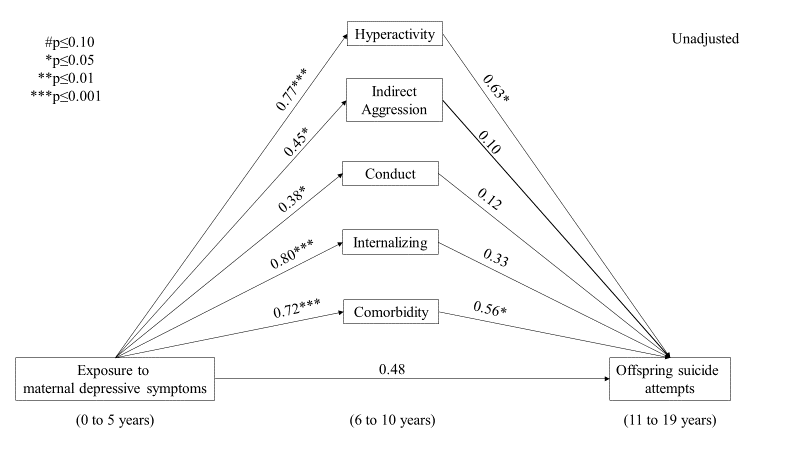


^a^Inverse probability weights were used to produce estimates that accurately reflect the characteristics of the Canadian population in 1994/1995 (the baseline of the longitudinal cohort from the NLSCY), excluding full-time members of the Canadian Armed Forces, inmates of institutions, and those residing (during the time of the survey) in Yukon, Nunavut, Northwest Territories and Indian reserves

Supplemental Figure 3. **Sensitivity analyses:** Adjusted^a^ beta coefficients of the total effects, and exposure, mediator associations, and mediator outcome associations with suicide-related thoughts as the outcome among offspring with cycle non-response, weighted^b^ to reflect the Canadian general population


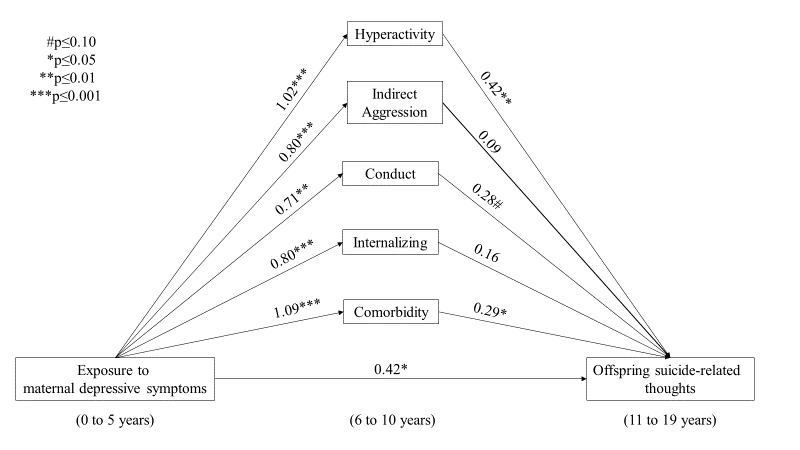


^a^Offspring age in years at baseline, offspring stressful life event (4-10 years), socio-economic status, maternal and paternal binge drinking (0-10 years), offspring sex, sex by exposure interaction

^b^Inverse probability weights were used to produce estimates that accurately reflect the characteristics of the Canadian population in 1994/1995 (the baseline of the longitudinal cohort from the NLSCY), excluding full-time members of the Canadian Armed Forces, inmates of institutions, and those residing (during the time of the survey) in Yukon, Nunavut, Northwest Territories and Indian reserves

Supplemental Figure 4. **Sensitivity analyses:** Adjusted^a^ beta coefficients of the total effects, and exposure, mediator associations, and mediator outcome associations with suicide attempts as the outcome among offspring with cycle non-response, weighted^b^ to reflect the Canadian general population


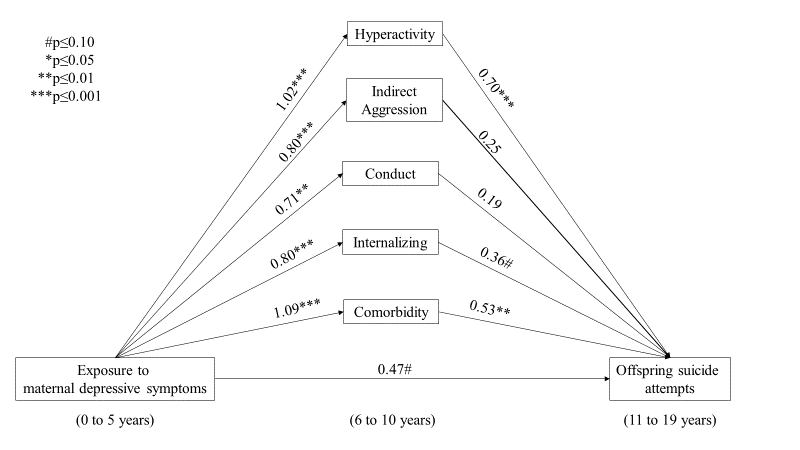


^a^Offspring age in years at baseline, offspring stressful life event (4-10 years), socio-economic status, maternal and paternal binge drinking (0-10 years), offspring sex, sex by exposure interaction

^b^Inverse probability weights were used to produce estimates that accurately reflect the characteristics of the Canadian population in 1994/1995 (the baseline of the longitudinal cohort from the NLSCY), excluding full-time members of the Canadian Armed Forces, inmates of institutions, and those residing (during the time of the survey) in Yukon, Nunavut, Northwest Territories and Indian reserves
